# Peripheral Tissue Homing Receptor Control of Naïve, Effector, and Memory CD8 T Cell Localization in Lymphoid and Non-Lymphoid Tissues

**DOI:** 10.3389/fimmu.2013.00241

**Published:** 2013-08-19

**Authors:** C. Colin Brinkman, J. David Peske, Victor Henry Engelhard

**Affiliations:** ^1^Department of Microbiology, Immunology, and Cancer Biology, Carter Immunology Center, University of Virginia School of Medicine, Charlottesville, VA, USA

**Keywords:** T cell trafficking, T cell memory, T cell heterogeneity, tumor infiltrating lymphocytes, T cell migration, T cell recirculation, memory T cell trafficking, effector T cell migration

## Abstract

T cell activation induces homing receptors that bind ligands on peripheral tissue vasculature, programing movement to sites of infection and injury. There are three major types of CD8 effector T cells based on homing receptor expression, which arise in distinct lymphoid organs. Recent publications indicate that naïve, effector, and memory T cell migration is more complex than once thought; while many effectors enter peripheral tissues, some re-enter lymph nodes (LN), and contain central memory precursors. LN re-entry can depend on CD62L or peripheral tissue homing receptors. Memory T cells in LN tend to express the same homing receptors as their forebears, but often are CD62Lneg. Homing receptors also control CD8 T cell tumor entry. Tumor vasculature has low levels of many peripheral tissue homing receptor ligands, but portions of it resemble high endothelial venules (HEV), enabling naïve T cell entry, activation, and subsequent effector activity. This vasculature is associated with positive prognoses in humans, suggesting it may sustain ongoing anti-tumor responses. These findings reveal new roles for homing receptors expressed by naïve, effector, and memory CD8 T cells in controlling entry into lymphoid and non-lymphoid tissues.

## Introduction

T cells are capable of assuming an impressive array of functional phenotypes. Much of this variation can be traced to differences in the place, context, or time since antigen exposure. The best recognized example of effector cell heterogeneity is the subset specialization of CD4 T cells based on cytokine secretion profiles (Th1, Th2, Th17, and Treg) (1). However, another example of functional specialization is the programing of CD4 and CD8 T cells to express different selectins, integrins, and chemokine receptors, which enable homing to different sites in the body. The particular constellation of such “homing receptors” expressed by individual cells depends on antigen encounter, and on microenvironmental characteristics of the secondary lymphoid organs (SLO). Just as importantly, however, the ability of such T cells to enter any particular tissue is dependent on which homing receptor ligands are expressed on the associated vasculature. Here we review the range of trafficking programs expressed by naïve, effector, and memory CD8 T cells, and the extent to which they dictate T cell entry into SLO and peripheral tissues, particularly tumors.

## T Cell Homing Receptor Heterogeneity during the Primary Response

T cell entry into tissues from the bloodstream is controlled by a multistep adhesion cascade involving interactions between homing receptors on the surface of T cells with their respective ligands on vasculature (2). Naïve T cells enter lymph nodes (LN) via L-selectin (CD62L) and chemokine receptor CCR7, which bind ligands on high endothelial venules (HEV) (3). Upon differentiation into effectors, CD62L and CCR7 are downregulated, and new homing receptors upregulated (Tables 1 and 2). Integrin α4β7 and CCR9 support homing to gut-associated tissue, the vasculature of which expresses the ligands MAdCAM-1 and CCL25 (4–7). In contrast, the ligands for E-selectin and P-selectin (ESL and PSL respectively) enable homing to skin, where inflamed vasculature expresses these selectins (8–11). While CCR4 is reported to be necessary for CD4 T cell entry into inflamed skin (12), other work has shown that CD4 and CD8 T cell infiltration does not require CCR4 and instead may depend on CCR10 (13), or CXCR3 and CCR5 (14). Much less is known about which homing receptors enable T cell entry into other tissues. α4β1 integrin, which binds VCAM-1, has been implicated in T cell infiltration into the brain (15, 16), lung (17), and bronchus-associated lymphoid tissue (18, 19). While activated CD8 T cells express many different chemokine receptors (Table 2), there is remarkably little direct information about the expression of their ligands in different tissues, which is essential in understanding the role they might play.

**Table 1:** **Adhesion molecules expressed by murine CD8 T cells**.

Adhesion molecule	Expression	Primary ligand/binding partner	Constitutive ligand expression	Inducible ligand expression	Ligand expression in tumor vasculature
α4β1	Low on naïve, upregulated upon activation	VCAM-1	Bone marrow, low levels in HEV	Inflamed brain, lung (BALT), liver	Sometimes detected
α4β7	Reported low on naïve, upregulated upon activation	MAdCAM-1	Mesenteric LN, Peyer’s patch HEV, small intestine postcapillary venules	Increased by inflammation, including in some sites beyond gut mucosa	Not known
αEβ7 (CD103)	None or low on naïve, upregulated on T cell subsets at epithelial surfaces	E-cadherin	Epithelia	N/A	Often downregulated during epithelial to mesenchymal transition
αLβ2 (LFA-1)	Present on naïve and activated T cells	ICAM-1	Postcapillary venules	Increased by inflammation	Frequently detected
CD44	Low on naïve T cells, upregulated upon activation	Hyaluronan	Connective, endothelial, neural tissue	Increased by tissue injury	Accumulates in many tumors
ESL	Absent on naïve T cells, upregulated upon activation	E-selectin (CD62E)	Low levels in dermal postcapillary venules	Inflamed dermal postcapillary venules, other inflamed postcapillary venules	Unclear, but blockade can reduce T cell infiltration
L-selectin (CD62L)	Naïve T, memory subsets, downregulated upon activation	Peripheral node addressin (PNAd)	LN HEV endothelial cells	Inflamed non-HEV blood endothelium	Subset of vessels in some tumors
PSL	Low on naïve T cells, upregulated upon activation	P-selectin (CD62P)	Low levels in dermal postcapillary venules	Inflamed dermal postcapillary venules, other inflamed postcapillary venules, activated platelets	Unclear, but blockade can reduce T cell infiltration

**Table 2:** **Chemokine receptors expressed by murine T cells**.

Receptor	T cell receptor expression	Ligand	Constitutive ligand expression	Inducible ligand expression	Ligand expression in tumor vasculature
CXCR3	Activated Th1, activated CD8	CXCL11 (ITAC)		Induced by Th1 inflammation	Not known
		CXCL10 (IP-10)	
		CXCL9 (MIG)	
CXCR4	Low to absent on naïve, upregulated after activation CD8 > CD4	CXCL12 (SDF-1)	Bone marrow endothelium, thymus, lung, lymphoid organs		Not known
CXCR6	Th1 activated CD8 T cells	CXCL16		Induced by Th1 inflammation	Induced by radiation
CCR1	Memory T cells	CCL3 (MIP-1a)		Induced by inflammation	Not known
		CCL5 (RANTES)	
		CCL7 (MARC)	
		CCL16 (LCC-1)	
CCR2	Subsets of CD4 and CD8 T cells, activation dependence unclear	CCL2 (MCP-1)		Induced by inflammation	Not known
		CCL7 (MARC)	
		CCL12 (MCP-5)	
CCR3	Th2 > Th1 activated CD8 T cells	CCL5 (RANTES)		Induced by inflammation	Not known
		CCL7 (MARC)	
		CCL8 (MCP-2)	
CCR4	Th2 *in vitro* activated CD8 T cells	CCL17 (TARC)		Induced by inflammation, particularly in dermis	Not known
		CCL22 (MDC)	
CCR5	Th1 activated CD8 T cells	CCL5 (RANTES)		Induced by inflammation	Not known
		CCL4 (MIP)	
		CCL3 (MIP)	
CCR6	Th17 activated CD8 T cells	CCL20 (MIP-3a)	Skin, intestinal villi	Upregulated in dermis after inflammation	Not known
CCR7	Naïve CD4, CD8 T cells, memory T cell subsets	CCL19 (MIP-3b)	Lymphoid organs		Not known
		CCL21 (SLC)	
		21-Leu periphery	
		21-Ser in LN	
CCR8	Subset of Th2 memory, negligible on CD8 T cells	CCL1 (TCA-3)		Induced by Th2 inflammation	Not known
CCR9	Subsets of naïve and activated CD4 and CD8 T cells	CCL25 (TECK)	Small intestine		Not known
CCR10	Skin-homing activated CD4 and CD8 T cells CD4 > CD8	CCL27	Skin	Upregulated in epidermis after inflammation	Not known

Expression of some homing receptors on effector T cells is determined by their activation site. CD4 T cells activated in cutaneous LN upregulate PSL, while those activated in mucosal LN upregulate α4β7 (20). This is mirrored *in vitro* using dendritic cells (DC) to activate CD8 T cells. DC from skin-draining LN induce ESL and PSL, while DC from mesenteric LN or Peyer’s patches induce α4β7 and CCR9 (21–23) based in part on their synthesis and presentation of retinoic acid (24, 25). However, α4β7 can be also induced without RA (23, 24, 26). Similarly, induction of CCR10 on human T cells is promoted by DC processing of Vitamin D3 to 1,25(OH)_2_D_3_, but this effect is less pronounced for mouse T cells (27). IL-2 and IL-12 are potent inducers of PSL expression on T cells *in vitro*, but dispensable *in vivo* (28, 29). *In vitro* studies have shown that induction of CCR5 on activated mouse CD4 and CD8 T cells requires IL-12 (30), while CXCR3 induction requires IFN-γ (31). Even less is known about the factors that control the induction of other homing receptors.

Recently, we examined homing receptor expression during CD8 T cell activation in different LN and spleen. Intravenous (IV) immunization with bone marrow derived DC activates T cells in mediastinal LN and spleen, most of which upregulate α4β1 integrin and PSL but not ESL or α4β7 (32–34). Intraperitoneal (IP) immunization activates T cells in mesenteric and mediastinal LN, which express α4β7 integrin and PSL (32, 33). Finally, subcutaneous (SC) immunization activates T cells in skin-draining LN, most of which express ESL and PSL, and some of which also express α4β1 (33). This work defines three major CD8 T cell effector populations that differentially express α4β7, α4β1, or ESL. Each of these molecules mediates the initial capture and tethering interaction of T cells with the vasculature (35–37), providing a basis for tissue selectivity, while α4β1 can also mediate firm adhesion (38). In contrast, expression of chemokine receptors shows little variation with activation site. Most activated CD8 T cells in all LN express CXCR3, and smaller subsets co-express CCR3, CCR4, CCR5, CCR6, and CCR9 (33). Only CCR9 expression varies significantly, with the largest fraction present on cells activated in mesenteric LN.

These results identify a previously unrecognized subset of effectors that uniformly expresses α4β1, but little ESL or α4β7, which is generated in the mediastinal LN and spleen by IV immunization. Other work has shown that IV immunization induces T cells that are incapable of mediating contact hypersensitivity (39), entering the gut (32), or controlling SC melanomas (40). Our work suggests that these observations reflect a homing receptor profile that does not enable T cell entry into skin or gut tissue. Conversely, as induction of α4β1 is weak after SC immunization, T cells generated by this route may only poorly infiltrate sites that require this integrin for entry. The layered coexpression of multiple chemokine receptors by CD8 T cells contrasts with a study that associated expression of CXCR3, CCR4, and CXCR5 with functionally distinct CD4 T cell subsets (41), but is consistent with another study showing coordinate expression of CCR4, CCR6, and CCR10 by human CD4 T cells (42). Thus, individual CD8 T cells may be more multipotential in their homing specificity than CD4 T cells. In any case, infiltration is ultimately dependent on expression patterns of the chemokines themselves, which remains somewhat poorly characterized (43). The multipotential chemokine-sensing capability of CD8 T cells may also provide a failsafe mechanism to ensure the entry of these effector cells into peripheral sites occupied by pathogens or tumors.

## CD8 T Cell Redistribution among LN

While some activated CD8 T cells leave SLO bound for inflamed peripheral tissues, others traffic to antigen-free LN (34, 44). These LN-redistributed cells resemble fully differentiated effectors by dividing extensively and secreting IFNγ (34). However, at least some were central memory precursors (34). LN redistribution depends in part upon residual expression of CD62L by some of these differentiated CD8 T cells (34). α4β7 integrin has long been known to enable activated T cell entry into mesenteric LN (34, 45). Recently, we found that activated CD8 T cells also redistribute into antigen-free LN using α4β1 and ESL (46); α4β1 enables entry into all LN, and ESL mediates selective entry into skin-draining LN. This results in differential accumulation of ESL^+^ and α4β1^+^ T cells in skin-draining vs. non-skin-draining LN after SC immunization or transfer of SC-primed effectors into naïve hosts. Others have shown that CD62Lneg T cells can enter inflamed, but not resting, LN using CXCR3 (47) or PSL (48). Thus, homing receptors normally associated with trafficking to peripheral non-lymphoid tissues also control the distribution of activated T cells among different lymphoid tissues, even in the absence of inflammation.

## Homing Receptor Expression and Regional Localization of T Cell Memory

Like effector cells, memory T cells are made up of distinct subsets. Central memory T cells (T_CM_) are defined as CD62L^+^CCR7^+^ and are found primarily in LN, while effector memory cells (T_EM_) are defined as CD62LnegCCR7neg and are found predominantly in peripheral tissue, spleen, and blood (49–52). Resident T_EM_ (rT_EM_) (52, 53) are retained permanently at epithelial surfaces, likely through expression of the E-cadherin receptor αEβ7, which is detected using anti-CD103 specific for the αE subunit (54–56). Migratory T_EM_ (mT_EM_) are CD103neg and recirculate (54, 57, 58). T_EM_ can express homing receptors associated with entry into peripheral tissue (54, 59–62).

Expression of CD62L and CCR7 and LN residence have been used somewhat interchangeably to define central memory cells (T_CM_) (49, 63). However, many memory cells in LN do not express one or both of these molecules (34, 64, 65). It has been proposed that mT_EM_ cells exit peripheral tissue through the afferent lymphatics (66, 67), and utilization of this pathway by CD4 T cells has recently been directly demonstrated (68). Because the afferent lymphatics drain into LN, mT_EM_ could be a component of what is generally thought of as T_CM_ despite their lack of CD62L expression. We found that CD62Lneg memory CD8 T cells in LN continue to express ESL, PSL, and α4β1 in patterns that mirror those of primary effectors (46). The distribution of these memory cells also reflects that of LN-redistributing effectors, with ESL^+^ memory CD8 T cells tending to reside in skin-draining LN, and ESLneg α4β1^+^ T cells tending to reside in non-skin-draining LN and spleen. Importantly, SC immunization, which induces ESL^+^ memory T cells, results in enhanced memory T cell residence in skin-draining LN and augmented recall responses to skin immunization challenge (46). Thus, the CD8 T cells we have identified share properties of both T_CM_ and T_EM_. They seem analogous to a recently described population of recirculating ESL^+^ memory CD4 T cells in skin and LN that do not express CD62L (68).

Interestingly, we found that these CD62Lneg LN-resident CD8 memory T cells can be reprogramed to express new peripheral tissue homing receptors, with minimal loss of those previously expressed (46). Thus, we have defined cells with enhanced representation in skin-draining LN, which expand upon rechallenge *in vivo*, and are plastic enough to be reprogramed to express new homing receptors. This is perhaps the best of both worlds in terms of host protection: enhanced regional memory as well as a systemic component that can be reprogramed. Thus T cell memory is comprised not only of cells that permanently reside in non-lymphoid tissue, and cells that almost exclusively recirculate among SLO, but also cells that recirculate between tissue and LN. These latter cells may include both classically defined mT_EM_ as well as cells that also have characteristics of T_CM_. CD103 is useful for distinguishing migratory and resident memory in peripheral tissues, and CD62L is useful for defining classical TCM. However, we lack phenotypic markers to distinguish LN-resident CD62Lneg subpopulations, and they currently must be studied by examining functional phenotype and migration. Vaccination strategies must consider the patterns of homing receptors induced by different immunization routes. These results also suggest that appropriate prime-boost regimens might be able to generate protective memory with multipotential homing capability.

## CD8 T Cell Homing to Tumors

While we have a good understanding of control of CD8 T cell infiltration into LN, skin, and gut, the requirements for entry into other tissues are poorly defined. Of particular interest is infiltration into tumors. Several studies have demonstrated that the presence of a CD8 T cell infiltrate in tumors is associated with a positive prognosis in human cancer patients (69–72). A panoply of homing receptors have been implicated in T cell infiltration in various tumor models, including LFA-1, α4β1, CD44, ESL/PSL, CXCR3, CCR2, CCR5 ((73–79), our unpublished observations). However, seemingly conflicting roles have been reported for LFA-1 ligand ICAM-1 (73, 74). In addition, chemokine CCL5 has been correlated with both positive and poor prognosis (80, 81). This may reflect differences in ligand expression in different tumor types, locations, or differential recruitment of additional cell populations. An important factor limiting T cell entry is the minimal expression of homing receptor ligands, including ICAM-1, E-selectin, and CXCR3 ligands on tumor vasculature. (82–84). Endothelin B receptor, CD73, and vascular endothelial growth factor (VEGF) have been shown to limit ligand expression (73, 85, 86). This is consistent with the overall poor infiltration of adoptively transferred effector T cells in murine and clinical studies (87–90). Conversely, inflammatory stimuli and radiation have been shown to enhance CD8 T cell entry through upregulation of homing receptor ligands (79, 83, 91) Thus, one approach to improve cancer immunotherapy is to identify and manipulate the expression of homing receptors and vascular ligands to enhance infiltration of CD8 effectors into tumors.

Although naïve T cells are generally excluded from peripheral tissues, we have found that they infiltrate and are activated in tumors of multiple tissue origins growing in the lungs, SC space, or peritoneal cavity (92). Naïve T cells infiltrate tumors by interacting with tumor associated vasculature that resembles that of LN HEV by expressing PNAd and CCL21, the ligands for CD62L and CCR7, respectively (Peske et al., manuscript submitted). While PNAd^+^ vessels are normally found only in LN, chronic inflammation induces their development in many peripheral organs, often in the context of accumulations of hematopoietic and stromal cells that organize into structures termed tertiary lymphoid organs (TLO) (93–95). PNAd^+^ vessels have also recently been identified in several human tumors, although it was not shown whether they were associated with TLO (96). Other studies have identified TLO in human tumors associated with CCL21 expression (97–99). PNAd expression on HEVs in LN and TLO is primarily controlled by signals through the lymphotoxin-beta receptor (LTβR) (94, 100–105). In contrast, we found that PNAd expression in tumors does not require LTβR signaling (Peske et al., manuscript submitted). Instead, effector lymphocytes induced the development of LN-like vasculature in part via secretion of IFNγ, which enhanced CCL21 expression. Thus, novel pathways control the development of HEV-like tumor vasculature. Importantly, HEV density or presence of TLO in human tumors correlates with positive prognosis (96, 97). The work of our lab and others suggests this is due to the recruitment of naïve T cells and subsequent generation of anti-tumor immune responses directly in the tumor (92, 106). Therefore, inducing HEV development in tumors may be a valuable therapeutic intervention.

## Conclusion

T cell homing to inflamed peripheral tissues is controlled by expression of homing receptors induced by activation that vary according to the route of immunization. Our and others’ work has built upon this understanding by illuminating surprising new roles for homing receptors expressed by naïve, effector, and memory CD8 T cells in controlling their entry into both lymphoid tissues and tumors. It remains to be seen whether additional homing receptors are involved in trafficking to regional LN, peripheral tissues other than skin and gut, and tumors in different body locations. A critical and still poorly described aspect is which homing receptor ligands are expressed by different tissues and tumors, and how this is positively and negatively regulated. It also is not clear how tissue-resident, lymphoid-resident, and migratory memory T cells interact to confer protection, or how to achieve an optimal mixture by vaccination. Finally, the notion of enhancing T cell immunity against localized pathogen infection or metastatic tumors growing in different locations by regional immunization to induce expression of appropriate homing receptors has yet to be incorporated into vaccine strategies. Nonetheless it is clear that T cell trafficking patterns are a source of both great specificity and flexibility waiting to be fully exploited for therapeutic benefit.

## Conflict of Interest Statement

The authors declare that the research was conducted in the absence of any commercial or financial relationships that could be construed as a potential conflict of interest.
